# Drug prescriptions preceding opioid-related deaths–a register study in forensic autopsy patients

**DOI:** 10.1371/journal.pone.0285583

**Published:** 2023-05-31

**Authors:** Jonatan Walde, Lisa Andersson, Björn Johnson, Anders Håkansson

**Affiliations:** 1 Umeå University and Region Västerbotten, Umeå, Sweden; 2 Faculty of Health and Society, Department of Social Work, Malmö University, Malmö, Sweden; 3 Lund University, School of Social Work, Lund, Sweden; 4 Department of Clinical Sciences Lund, Lund University, Psychiatry, Lund, Sweden; 5 Region Skåne, Malmö Addiction Center, Malmö, Sweden; University of Rome La Sapienza Sapienza Faculty of Medicine and Dentistry: Universita degli Studi di Roma La Sapienza Facolta di Medicina e Odontoiatria, ITALY

## Abstract

**Background/Aim:**

Opioid overdose deaths have increased in Sweden and other developed countries in recent decades, despite increased treatment efforts and harm-reduction interventions. Further knowledge in this field is needed if this trend is to be reversed. Previous research suggests that mental health and patterns of prescription of opioids and other prescription drugs are associated with increased opioid-related mortality. The present study therefore aimed to investigate what drugs were prescribed during the last six months of life to individuals with a history of illicit substance use who died with opioids present in their blood, the relationship between drugs prescribed and drugs found in blood at time of death, and if prescription of specific drugs was temporally associated with death.

**Methods:**

This was a retrospective, register-based observational study that utilized data from the National Board of Forensic Medicine, the Prescribed Drug Registry, regional health care services, and municipal social services. We used conditional logistic regression to find temporal associations between the prescription and dispensing of drugs and time of death.

**Results:**

Prescription and dispensing of alprazolam and diazepam were temporally associated with death. The most frequently dispensed drugs were zopiclone, pregabalin, methylphenidate, diazepam and oxycodone. Methadone, alprazolam, and buprenorphine were the drugs most often found in the blood. Opioids and tranquilizers in combination were found in a vast majority of deaths, and prescription data suggested that the use of these drugs was illicit in a majority of cases.

**Conclusion:**

Prescription of certain drugs, especially alprazolam and diazepam, should be made with great caution to patients with a history of illicit substance use or concurrent use of opioids.

## 1. Introduction

Drug-related deaths, with opioids involved in a large majority of cases, are an increasing problem in developed countries, sometimes referred to as an opioid crisis or epidemic. According to a recent report from the OECD (The Organization for Economic Co-operation and Development), the average number of drug-related deaths per capita in OECD countries increased by 20% between 2011 and 2016. The highest rate of drug-related deaths in 2016 among the OECD countries were observed in the US, Canada, Estonia, Sweden and Norway, in that order [1]. A link has been established between opioid prescribing, illicit opioid use, and overdose deaths [2,3]. In Sweden, the annual incidence of at least one opioid prescription has slightly decreased in recent years, but the yearly incidence of prescription of oxycodone has increased markedly [4]. The OECD report addresses possible explanations for the increased number of opioid-related deaths, such as changed patterns of prescription and a growing illicit market [1], but these topics will not be covered in this article.

The mental health of patients is likely to affect opioid prescribing. In the US, patients with a mental disorder are prescribed more opioids than patients without a mental disorder when controlling for multiple factors such as the cause and severity of pain [5]. Other studies have shown that untreated psychiatric comorbidities increase the risk of opioid addiction and opioid-related mortality among patients prescribed opioids [6,7].

In addition to opioids, other prescription drugs used in combination with opioids increase the risk of opioid-related death. In particular, the use of benzodiazepines (BZD), but also the use of z-drugs (zopiclone, zolpidem) and pregabalin, is widespread among opioid-addicted individuals, often involved in opioid deaths, and seems to be associated with increased mortality in combination with opioids both when prescribed and used illicitly [7–26]. An American study on veterans who were prescribed opioids concluded that simultaneous BZD prescription increased the risk of overdose death in a dose-response manner. In addition, patients who had a previous but not ongoing BZD prescription were at greater risk of overdose death compared to patients who had never received BZD [9]. Another study found the rate of overdose deaths to be ten times higher in patients who were prescribed both opioids and BZD compared to patients who were prescribed opioids only [10]. The risk of opioid overdose has also been shown to increase with the length of BZD treatment [7]. Similarly, a Swedish study found the prescription of z-drugs and pregabalin to be associated with increased mortality in opioid substitution treatment (OST) [13]. Despite the increased mortality among opioid users when prescribed BZD, a New Zealand study found that BZD prescription was relatively more frequent in patients with an opioid addiction compared to both the general population and patients addicted to other substances [15]. A study from the US found that the overdose risk in patients receiving concurrent treatment with opioids and BZD increased if more than one prescriber was involved in the treatment [24]. Another recent Danish study found that BZD are prescribed inappropriately often to patients addicted to drugs or alcohol, and that patients addicted to opioids received three times more BZD compared to patients addicted to other substances. This study also found that patients with addiction who were treated in specialized psychiatric care were less likely to receive high doses of BZD, and that general patterns of prescription indicated inappropriate use to a larger extent than treatment for psychiatric disorders [16]. In addition to the widespread prescription of BZD, illicit use of BZD in patients with nonmedical use of opioids is frequent [17,18,27]. Studies on self-perceived motives for BZD use and related behavior have found that self-therapeutic use of BZD in this group is more common than hedonic use, but in a majority of cases the same individual experienced both motivations at different times. Hedonic use was more associated with buying BZD on the black market, parenteral administration, and using BZD in combination with other drugs, such as opioids [17,18,27]. Problematic use of BZD among patients in buprenorphine treatment for opioid addiction has been found to be associated with impaired quality of life, mental and physical health problems, and a difficult living situation [19]. BZD use has also been linked to poorer outcome in methadone treatment for opioid addiction, with more urine samples positive for opioids during on-going treatment [28].

Despite extensive treatment efforts and increasing harm-reduction interventions, opioid-related deaths have increased in Sweden over time [29]. The National Board of Health and Welfare (NBHW) recommends that counties in Sweden implement OST, syringe-exchange programs, and recently also naloxone programs. The number of counties that have adopted these interventions is increasing, but only OST is implemented nationwide [30]. During the ten years following 2006, the number of fatal drug intoxications in Sweden more than doubled [29]. Approximately 90% of the drug-related deaths are caused by opioids [30]. The reason for the rise in opioid-related mortality in Sweden and other countries is presently not known. Increased knowledge in this relatively unexplored field is key if this trend is to be halted and ultimately reversed. The present study therefore aimed to investigate what events precede opioid-related deaths, with focus on drug prescriptions and drugs found in blood at the time of death. We sought to answer the following four questions:

1. What drugs were prescribed during the last six months of life to individuals with a history of illicit drug use who had opioids present in their blood at the time of death?
2. What drugs were found in the blood at the time of death?
3. To what degree were the drugs found in the blood at the time of death prescribed?
4. Were any drugs or drug classes associated with prescription during the last month of life in this population?

## 2. Methods

### 2.1 Study design

This was a retrospective, register-based observational study that utilized data from The National Board of Forensic Medicine (NBFM) and The Prescribed Drug Register (PDR). The study was approved by the Regional Ethics Committee in Lund, Sweden (case no. 2014/547, 2015/369 and 2016/771). As the study involved only deceased subjects, no informed consent procedure was carried out.

### 2.2 Setting

Every year, a little over 90,000 people die in Sweden [31]. Of these, approximately 6% undergo a forensic autopsy [32]. Forensic autopsies, including toxicological screenings, are done upon request from the police or a prosecutor in cases where the cause of death is unknown or if the person has died under unclear circumstances [33,34]. Previous work has suggested that forensic autopsies are carried out in over 90% of drug-related deaths in Sweden [35,36].

### 2.3 Study population

This study utilized a cohort selected for previous studies on opioid-related mortality [37,38]. The selection of the cohort and collection of data not related to drug prescriptions was done by L.A. The study population included all deaths in Skåne county, Sweden, during a four-year period who underwent a forensic autopsy, where an opioid was found at the forensic chemical analysis, and where there was indication of illicit substance use, regardless of the cause of death. Individuals over 64 years of age at the time of death were excluded, since opioid overdoses and opioid dependence are rare among people 65 years and older in Sweden [39,40]. Skåne County has approximately 1.4 million inhabitants. The material consisted of two time periods: January 1, 2012 to December 3, 2013 and July 1, 2014 to June 30, 2016. The division into these two time periods was done to make it possible to, in previous studies on the same cohort, compare the deaths in the two periods. The first period is prior to 2014, when OST in Skåne was made more accessible, and the second period includes deaths that occurred after this change [35,37].

To identify cases where opioids were present in the body at the time of death, forensic reports at The National Board of Forensic Medicine (NBFM) in Lund, Sweden, were inspected manually. The first selection included all deaths that had occurred in Skåne where the person was also registered as living in Skåne in the national population registry. Approximately 1000 forensic autopsies are performed at NBFM in Lund each year. This includes all deaths in Skåne as well as deaths in one neighbouring small county and the southern parts of two additional counties. Opioids were present in 503 out of the roughly 4,000 cases during the above-mentioned time periods. The forensic reports were read to determine if there was a patient history of illicit substance use. A person was considered to have a history of illicit drug use if the report from the forensic autopsy or the police report included in the NBFM journals mentioned use of one or more narcotic substances, if the autopsy report mentioned needle marks, or if illegal drugs (e.g. THC, amphetamine or cocaine) were found at the forensic chemical analysis. In cases where such a history was found, additional information was collected from NBFM and The Prescribed Drug Register (PDR), see data sources below. Information was also collected from health care, municipal social services and the Prison and Probation Service, but these data are not used in the present study. We refer to a previously published article for detailed descriptions of these authorities and for background information [37].

The cause of death was determined using information stated in the report from the forensic autopsy. Cases were categorized as “opioid overdose deaths” if the cause of death was unintentional opioid overdose, intentional opioid overdose (suicide) or intoxication with opioids with unknown intent. All other causes of death were categorized as “non-opioid overdose deaths”. Unclear cases were discussed with a forensic pathologist and associate professor at NBFM in Lund. The vast majority of cases were intoxications with several substances.

### 2.4 Data sources and variables

Data were collected from NBFM and PDR and the data sources were linked using the personal identification number, which is unique to each Swedish citizen. The data from NBFM include personal identification number, sex, age, registered address in population registry, place of death, and cause of death for each subject. The toxicological data include the prevalence and concentration of prescription drugs, alcohol, and illegal drugs. Information about all prescribed drugs for each individual in their last six months of life was obtained from PDR, which is a nationwide database containing information on all prescribed drugs dispensed at Swedish pharmacies since July 1, 2005. The data from PDR include date of prescription, date of dispensing, Anatomical Therapeutic Chemical classification system (ATC) code, trade name, package size and dosage forms for each time that a prescription drug was dispensed. Drugs that were prescribed but not dispensed, and drugs that the individual received directly without a prescription (e.g. during in-patient care), are not in the registry and therefore not included in our study. Information about ongoing OST was retrieved from searches in health care journals, as patients in OST often receive their medication at the OST clinics without necessarily having a prescription in PDR for methadone or buprenorphine.

### 2.5 Statistical analysis and data management

Information from NBFM, health care, municipal social services and the Prison and Probation Service was linked together in a SPSS file by L.A. Information about drug prescriptions was merged with this file. All data management and statistical analysis regarding drug prescriptions was performed by author J.W. SPSS version 25 was used for data management and analysis. Conditional logistic regression was applied to calculate if the prescription of certain drugs increased the odds of death during the same month. This was done by determining whether each individual had at least one prescription for a given drug during each of the last six months of their life, creating a binary variable for prescription of each drug of interest per month (see below). The last month in life becomes the case and the five previous months become the control for each individual. By using conditional logistic regression and conditioning the analysis on each individual, each individual becomes his or her own control as in a case-crossover design. The same procedure was used to analyze the dispensing of the same drugs. Since there is no pre-set command for conditional logistic regression in SPSS, the analysis was run using the pre-set command for Cox-regression in the survival package in SPSS, but with the time variable set to one for all cases and controls.

To find the frequency of prescribed drugs and the relationship between drugs prescribed during the last six months of life and drugs found in blood at time of death, information from the forensic examination and chemical analysis was merged with data from PDR. For each prescription two variables were created: one representing the difference between date of death and date of prescription, and one representing the difference between date of death and date of dispensing in months. Two separate files were created: one containing all individual prescriptions issued during the last six months of life, and one containing all individual prescriptions dispensed during the last six months of life. This was done to account for drugs prescribed more than six months prior to death but dispensed within six months of the date of death. The total number of dispensed prescriptions for each drug during the last six months was acquired through the descriptive statistics package, but no calculations were made in this regard. All ATC codes were then, using a pre-set function in SPSS, given a number in a new variable based on alphabetical and numerical order of ATC codes occurring in our material. The number of months between prescription and death for each individual prescription was then multiplied by 1000 and added to the number replacing the ATC code. This produced a new variable containing information about both the number of months between prescription and time of death and the specific substance for each individual prescription. Rearranging the file so that all prescriptions to the same individual were on the same row (so called wide format) then made it possible for SPSS to count prescriptions of specific drugs issued during each of the last six months of life for each individual. The resulting variables were then converted into binary format, and the same procedure was repeated for drugs dispensed during the last six months of life. These resulting variables were then used to perform the conditional logistic regression described above.

The same principle used to determine if a drug was prescribed during each of the six months prior to death was also used to determine if each individual had a certain drug prescribed at least once during the last six months of life. If a person had at least one prescription of a substance found in blood at the time of death during the last six months of life, this substance was considered to possibly have been prescribed. Conversely, a drug found in blood at the time of death in individuals who did not have a single prescription of this substance during the last six months the substance was considered likely to be illicit, with the exception of the OST drugs methadone and buprenorphine. These drugs were considered likely to be illicit if the person, according to patient files, did not have ongoing OST at the time of death.

### 2.6 Drugs of special interest

A selection of drugs and drug classes was made for analysis in the conditional logistic regression, as it was neither possible nor desirable to analyze every substance that occurs in the material. As mentioned in the introduction, psychiatric comorbidities and the prescription of opioids, BZD, z-drugs and pregabalin are associated with increased opioid-related mortality. Therefore, opioids, BZD, pregabalin, z-drugs, antipsychotics, stimulants, and antidepressants were included in the analysis. All drugs as one group, all drugs that affect the nervous system as one group, and all somatic drugs as one group were analyzed to control for general patterns not unique to a certain substance.

## 3. Results

### 3.1 Description of the study population

The study population consisted of 235 individuals, 186 male and 49 females, between the ages of 18 and 64, where one or more of the opioids heroin, methadone, buprenorphine, fentanyl, oxycodone, or morphine were detected in forensic toxicological screenings. The mean age at the time of death was 38 years. In 82% of the cases, opioid overdose was the underlying cause of death. Of these, the opioids that caused the most overdose deaths were, in falling order, were methadone (44%), heroin (21%), buprenorphine (16%), fentanyl (15%), oxycodone (4%), and morphine (1%). In 18% of all cases in the cohort, the above-mentioned opioids were present in the blood, but opioid intoxication was not the main cause of death (i.e. due to somatic disease, intoxication from other substances, accident, or homicide). In the latter group, morphine and methadone were the most prevalent opioids, each accounting for approximately one third. In the vast majority of the 235 cases, other drugs besides the opioids mentioned above were also detected at the autopsy. Sixteen percent had ongoing OST at the time of death.

### 3.2 Prevalence of prescribed drugs

The five most frequently dispensed drugs in this population during the last six months of life were, in falling order, zopiclone (457 dispensed prescriptions), pregabalin (230), methylphenidate (219), diazepam (183), and oxycodone (154). The five drugs that were prescribed to the largest number of individuals during the last six months of life were, in falling order, zopiclone (70 individuals), mirtazapine (38), paracetamol (38), alimemazine (35), and pregabalin (34). Zopiclone alone accounted for 10.3% of all dispensed prescriptions and was prescribed in 30% of cases. Opioids, not including OST drugs, were prescribed to 51 individuals. Fifty-three individuals (23%) had no dispensed prescriptions during their last six months of life. In the group that had dispensed at least one drug during the last six months of life, the number of dispensed prescriptions ranged from 1 to 281. The first quartile was 5, the median was 11, and the third quartile was 29. See Table 1 for all prescriptions during this time period categorized by ATC-group and Table 2 for the 30 most prescribed drugs during the last six months of life.

**Table 1:** Total number of prescriptions in each ATC group dispensed during the last six months of life for the entire study population.

ATC-groups	Frequency	Percent
N—Nervous system	2572	75.5
R—Respiratory system*	236	6.9
A—Alimentary tract and metabolism	153	4.5
J—Antiinfectives for systemic use	125	3.7
C- Cardiovascular system	120	3.5
B—Blood and blood forming organs	61	1.8
M—Musculo-skeletal system	46	1.4
D—Dermatologicals	29	0.9
H—Systemic hormonal preparations, excluding sex hormones and insulins	26	0.8
G—Genito- urinary system and sex hormones	11	0.3
S—Sensory organs	11	0.3
Y—Non drugs, other	9	0.3
P—Antiparasitic products, insecticides and repellents	3	0.1
Total	3402	100

*ATC group R contains 17 dispensed prescriptions for opiates for cough relief and 70 for Promethazine prescribed not primarily as an antihistamine for allergy but as a sedative.

**Table 2:** The 30 most dispensed drugs during the last six months of life for all causes of death.

	Drug	Number of dispensions the last 6 months of life	Number of individuals with a prescription during the last 6 months of life	ATC-code	Percent of total number of filled prescriptions
1	Zopiclone	457	70	N05CF01	10.3
2	Pregabalin	230	34	N03AX16	5.2
3	Methylphenidate	219	20	N06BA04	4.9
4	Diazepam	183	27	N05BA01	4.1
5	Oxycodone	154	20	N02AA05	3.5
6	Propiomazine	138	25	N05CM06	3.1
7	Paracetamol	127	38	N02BE01	2.9
8	Olanzapine	123	10	N05AH03	2.8
9	Alprazolam	113	23	N05BA12	2.5
10	Oxazepam	108	25	N05BA04	2.4
11	Zolpidem	106	23	N05CF02	2.4
12	Mirtazapine	98	38	N06AX11	2.2
13	Alimemazine	95	35	R06AD01	2.1
14	Sertraline	92	18	N06AB06	2.1
15	Codeine—in combination with non-opioid analgesic	92	21	N02AA59 N02AJ06-9	2.1
16	Promethazine	70	22	R06AD02	1.6
17	Tramadol	69	18	N02AX02	1.5
18	Clonazepam	65	6	N03AE01	1.5
19	Omeprazole	64	22	A02BC01	1.4
20	Morphine	50	7	N02AA01	1.1
21	Hydroxyzine	41	14	N05BB01	0.9
22	Salbutamol	40	8	R03AC02	0.9
23	Gabapentin	39	4	N03AX12	0.9
24	Quetiapine	37	15	N05AH04	0.8
25	Duloxetine	37	5	N06AX21	0.8
26	Busprione	32	4	N05BE01	0.7
27	Sumatriptan	31	6	N02CC01	0.7
28	Venlafaxine	31	11	N06AX16	0.7
29	Propanolol	30	9	C07AA05	0.7
30	Acamprosate	30	4	N07BB03	0.7

### 3.3 Prevalence of drugs in blood at time of death

More than one opioid was present in the blood at time of death in 23% of opioid overdose deaths, but only in 2% of deaths from other causes. The number of substances, including metabolites, found in the blood at time of death ranged from 1 to 17, with a mean of 6. At least one BZD was present in the blood at time of death in 72% of opioid overdose deaths and in 71% of deaths from other causes. BZD, z-drugs, or pregabalin was present in 83% and 76% of cases respectively.

The five most prevalent drugs in blood at time of death were methadone (104 individuals), alprazolam (101), buprenorphine (59), clonazepam (57), and pregabalin (54). Alcohol was present in blood at time of death in 90 cases. To avoid including cases where alcohol was produced post mortem we used a cut-off at a blood alcohol concentration of > 0.5 ‰, which led to 40 cases where alcohol above this concentration was found [35]. The five most prevalent non-opioid prescription drugs were alprazolam (101), clonazepam (57), pregabalin (54), zopiclone (51), and diazepam (43). The five most prevalent drugs that can only be obtained illegally in Sweden were THC (41), heroin (35), cocaine (15), flubromazolam (5), and acetylfentanyl (4). Amphetamine was present in 32 cases but is not on the list of illegal drugs since it can be prescribed. See Table 3 for the prevalence of drugs in blood.

**Table 3:** Prevalence of drugs in blood at time of death.

Drug	All	Opioid overdose deaths	Non- opioid overdose deaths	All percent	Opioid overdose deaths percent	Non- opioid overdose deaths percent
Methadone	104	90	14	44	47	33
Alprazolam	101	87	14	43	45	33
Buprenorphine	59	52	7	25	27	17
Clonazepam	57	46	11	24	24	26
Pregabalin	54	47	7	23	24	17
Zopiclone	51	44	7	22	23	17
Codeine	48	43	5	20	22	12
Nordazepam*	48	36	12	20	19	29
Diazepam	43	32	11	18	17	26
THC	41	38	3	17	20	7
Heroin	35	35	0	15	18	0
Fentanyl total	35	31	4	15	16	10
Amphetamine	32	20	11	13	10	26
Morphine	29	16	13	12	8	31
Fentanyl	28	24	4	12	12	10
Oxycodone	21	12	9	9	6	21
Methylphenidate	17	13	4	7	7	10
Oxazepam	15	12	3	6	6	7
Tramadol	14	11	3	6	6	7
Benzoylecgonine (Cocaine)	12	10	2	5	5	5
Flunitrazepam	5	3	2	2	2	5
Nitrazepam	5	5	0	2	3	0
Flubromazolam	5	5	0	2	3	0
Acetylfentanyl	4	4	0	2	2	0
Bromazepam	3	3	0	1	2	0
Acrylfentanyl	2	2	0	1	1	0
Zolpidem	2	1	1	1	1	2
Spice	2	2	0	1	1	0
MDMA	2	1	1	1	1	2
Furanylfentanyl	1	1	0	0	1	0
Hydrocodone	1	0	1	0	0	2
Ethylmorphine	1	0	1	0	0	2
Opioids	235	193	42	100	100	100
> 1 opioid	45	44	1	19	23	2
Tranquilizers**	193	161	32	82	83	76
Benzodiazepines	169	139	30	72	72	71
Alcohol***	40	33	7	17	17	17
Antidepressants	67	49	18	29	25	43
Stimulants	62	44	18	26	23	43

* Nordazepam is a metabolite of diazepam and not registered as a drug in Sweden.

**At least one BZD, z-drug or pregabalin.

*** Blood concentration > 0.5 ‰.

### 3.4 Relationship between drugs found in blood at time of death and prescribed drugs

A majority of prescription drugs found in the subjects’ blood at the time of death were not prescribed to these individuals during the last six months of life. In the 57 cases where clonazepam or metabolites of clonazepam were found in the blood at the time of death, only two had a dispensed prescription for clonazepam during the last six months of life. Thus, the fraction of clonazepam use that is likely to be illicit is 97%. The five prescription drugs that had the highest fraction of use that is likely to be illicit were clonazepam (97%), codeine (90%), fentanyl (90%), buprenorphine (83%), and morphine (83%). Cases where heroin was present have not been included in the morphine group. The five drugs most frequently found in the blood at time of death with no prescription filled during the last six months of life were alprazolam (83), methadone (81), clonazepam (55), buprenorphine (49), and codeine (43). See Tables 4 and 5 for a complete list of the relationship between drugs found in blood at the time of death and prescription of the same drugs during the last six months of life.

**Table 4:** Relationship between drugs found in blood at time of death and drugs being prescribed during the last six months of life.

Drug	Cases with drug in blood at time of death	Cases with the drug prescribed during the last six months of life	Cases with drug in blood at time of death and the drug prescribed during the last six months of life	Cases with drug in blood at time of death but no prescription during the last six months of life	Percent of cases with drug in blood and no prescriptions during the last six months of life	Percent of cases with drug in blood and at least one prescription during the last six months of life
Opioids***	235	51	51	184	78%	22%
Heroin	35	0	0	35	100%	0%
Morphine*	29	7	5	24	83%	17%
Codeine	48	21	5	43	90%	10%
Methadone	104	23	23	81	78%	22%
Buprenorphine	59	11	10	49	83%	17%
Fentanyl	28	3	3	25	89%	11%
Akrylfentanyl	2	0	0	2	100%	0%
Acetylfentanyl	4	0	0	4	100%	0%
Furanylfentanyl	1	0	0	1	100%	0%
Oxycodone	21	20	9	12	57%	43%
Tramadol	14	18	3	11	79%	21%
Benzodiazepines	169	69	59	110	65%	35%
Alprazolam	101	23	18	83	82%	18%
Clonazepam	15	6	2	13	87%	13%
7-amino-clonazepam	57	6	2	55	97%	4%
Oxazepam	15	25	4	11	73%	27%
Diazepam	43	27	18	25	58%	42%
Nitrazepam	5	2	2	3	60%	40%
Flunitrazepam	5	3	1	4	80%	20%
Bromazepam	3	0	0	3	100%	0%
Flubromazolam	5	0	0	5	100%	0%
Pregabalin	54	34	28	26	48%	52%
Zopiclone	51	70	27	24	47%	53%
Zolpidem	2	23	2	0	0%	100%
Antidepressants	67	70	49	18	27%	73%

*Morphine and 6-monoacetylmorphine are two major metabolites of heroin, why cases with both 6-monoacetylmorphine and morphine present in blood have been excluded from the morphine group and regarded as heroin only.

** Number of subjects in OST treatment at time of death are presented for methadone and buprenorphine.

*** Excluding buprenorphine and methadone.

**Table 5:** OST treatment in relation to OST drugs, methadone or buprenorphine, found in blood at time of death.

	Yes	No	Missing
OST at time of death, from patient medical records*	38	195	2
OST drugs in blood at time of death, total	149	86	0
OST at time of death, from patient medical records, and OT drugs in blood at time of death	37	196	2
OST drugs in blood at time of death but no ongoing OST treatment	110	123	2
OST treatment during the last year of life including at time of death	55	178	2
OST treatment during the last year of life including at time of death and OST drugs in blood at time of death	47	186	2
OST treatment during the last 6 months of life including at time of death	51	182	2
OT treatment during the last 6 months of life including at time of death and OST drugs in blood at time of death	46	187	2
Methadone treatment at time of death	27	206	2
Buprenorphine treatment at time of death	11	222	2

### 3.5 Prescription drugs as a predictor of death

The case-crossover analysis for drugs prescribed and dispensed for each month in the last six months of life found statistically significantly increased odds of death within the same month for prescription of alprazolam (OR 9.9, 95% CI: 1.7–57.5) and diazepam (5.7, 1.1–30.1), as well as for dispensing of alprazolam (12.4, 2.1–74.8) and diazepam (7.5, 1.2–47.0). No other drugs or drug classes demonstrated a significant temporal association with death. See Tables 6 and 7 for the results from the conditional logistic regression.

**Table 6:** Prescription drugs as a predictor for overdose death, prescribed drugs. Results of conditional logistic regression.

Prescribed drugs	p	OR	95,0% CI for OR	
			Lower	Upper
Alprazolam	0.01	9.91	1.71	57.48
Diazepam	0.04	5.71	1.06	30.83
All drugs	0.10	0.47	0.16	1.15
Antidepressants	0.12	0.57	0.27	1.15
Clonazepam	0.13	5.91	0.59	59.38
Stimulants	0.16	0.44	0.14	1.40
Benzodiazepines	0.24	0.36	0.07	1.95
Z-drugs	0.25	1.43	0.78	2.62
Drugs that affect the nervous system	0.38	1.50	0.60	3.76
Pregabalin	0.40	1.44	0.62	3.31
Opioids	0.44	0.74	0.34	1.59
Somatic drugs	0.94	0.98	0.56	1.71
Oxazepam	0.96	1.06	0.13	8.52
Antipsychotics	0.96	1.03	0.41	2.60

**Table 7:** Prescription drugs as a predictor for overdose death, dispensed drugs. Results of conditional logistic regression.

Dispensed drugs	p	OR	95,0% CI for OR	
			Lower	Upper
Alprazolam	0.01	12.41	2.06	74.76
Diazepam	0.03	7.45	1.18	47.02
Antipsychotics	0.06	2.18	0.98	4.85
Clonazepam	0.06	15.94	0.90	281.01
Drugs that affect the nervous system	0.09	2.20	0.88	5.52
All drugs	0.09	0.46	0.18	1.14
Antidepressants	0.12	0.63	0.36	1.12
Benzodiazepines	0.12	0.25	0.043	1.43
Z-drugs	0.12	1.63	0.88	3.03
Stimulants	0.15	0.46	0.16	1.31
Pregabalin	0.46	1.40	0.57	3.41
Oxazepam	0.73	0.70	0.09	5.22
Opioids	0.74	0.88	0.43	1.84
Somatic drugs	0.91	1.03	0.61	1.73

## 4. Discussion

We have found that the drugs most prescribed during the last six months of life to subjects with a history of illicit substance use who die with at least one opioid present in their blood are analgesics, tranquilizers, antidepressants, antipsychotics and stimulants, suggesting high rates of polydrug use and psychiatric comorbidity. It is worth noting that the five most dispensed drugs in this population all have potential for misuse, especially in populations with a previous history of addiction [11,14,20,21,41–45]. The use of BZD, z-drugs and pregabalin has great misuse potential [26] and has been shown to increase opioid-related mortality [6–11,13,20] and at least one of these drugs was present in the blood at the time of death in a vast majority of cases and they were among the drugs most frequently dispensed by this population in our material. Previous research suggests that individuals with opioid addiction are prescribed BZD in excessive amounts [15,16] and it is possible that excessive prescription of these drugs occurs in our material as well. The widespread use of these substances, prescribed and illicit, in our material indicates a high demand for these drugs in the study population, but says nothing of the motives for using them. Previous studies found BZD to be frequently used as self-treatment among individuals with an opioid addiction, but they were also used recreationally to increase the high of opioids. The use of BZD in combination with opioids in patients addicted to opioids was associated with hedonic use to a larger extent than with self-treatment, and a clear majority of the patients who used illicit BZD did not limit their use to self-treatment [17,18].

The drug class most often found in blood in our material, aside from opioids where presence was an inclusion criteria, was benzodiazepines. While most benzodiazepines and opioids are available as prescription drugs, few individuals with these drugs in their blood at time of death had filled a single prescription for them during the last six months of life. Methadone and buprenorphine, both OST drugs used in Sweden, caused a majority of opioid overdose deaths, with methadone causing the largest number of deaths in our study. A large majority had used these drugs illicitly, as only one in six received OST at the time of death. This is consistent with previous findings [12,40]. The findings discussed above suggest that OST drugs are relatively easy to obtain outside of treatment [46], and the high number of deaths caused by OST drugs in our study is cause for serious concern. Illicit use has been found to be common among heroin and polydrug users who use these drugs both as self-treatment for their addiction and to achieve euphoria, but use is uncommon in more inexperienced opioid users [47–49]. Tre reasons for the increase in overdose deaths caused by methadone and buprenorphine [50] are beyond the scope of the present study, but is a problem that needs to be addressed by society and in future research. A previous study, based on the same data set as in this present study, showed that increased access to OST was not associated with an increase in opioid overdose deaths, nor with an increase in overdose deaths specifically related to methadone or buprenorphine [38].

The present study found a temporal association between death and the prescription and dispensing of alprazolam and diazepam. Alprazolam and diazepam were frequently found in blood at the time of death where alprazolam was relatively more prevalent in overdose deaths and diazepam was relatively more prevalent in non-overdose deaths. Alprazolam is regarded to be a drug with high misuse potential and more severe withdrawal symptoms compared to other BZD [51]. In all drug-related deaths in the US between 2010 and 2014, alprazolam was consistently among the top five most mentioned specific drugs on death certificates and diazepam was consistently in the top ten on the same list. No other BZD was in the top ten list presented [22]. Relative to other BZD alprazolam has been shown to be more toxic, resulting in longer lengths of stay at hospitals and higher frequency of admittance to intensive care units in cases of intoxication when controlled for dose, age, gender, co-ingested drugs and time to ingestion [52]. In addition, alprazolam has been shown to be the BZD involved in the most visits to emergency departments in the US related to drug misuse, both in absolute numbers and per prescription [49,53,54]. In a recent study, alprazolam prescription showed to be the single best predictor of opioid drug death in Michigan, US [23]. However, our study did not find BZD as a group to be associated with prescription during the last month of life. On the contrary, oxazepam was dispensed and prescribed to a lesser extent in the last month of life, though this was not statistically significant, indicating that oxazepam is a safer drug to use relative to alprazolam and diazepam.

The large proportion of individuals in our material who had alprazolam or diazepam in their blood at the time of death but did not have a prescription for these drugs during the last six months of life suggests that obtaining these drugs through means other than a prescription is relatively easy. However, our study also found that prescription and dispensing of these drugs was temporally associated with death. This finding can be interpreted in two ways. Firstly, the prescription of alprazolam and diazepam could indicate a worsening of mental health shortly before death, which could be independently associated with an increased risk of overdose and death. Secondly, it could be interpreted as an indication that these drugs are dangerous and have a direct effect on mortality. Some support for both of these interpretations can be found [6,7,10,17–19,55,56].

Among strengths of the present study, it can be noted that our study population included all deaths in a geographically defined area that met our criteria. Since the NBFM files were reviewed manually, selection was likely more accurate than if a search function had been used. In addition, the PDR lists all drugs dispensed at Swedish pharmacies since 2005. Taken together this means that the data presented in this study are likely to be very accurate.

The present study also has limitations. The study is a retrospective observational study, and therefore the results cannot be interpreted as causal relationships. The statistical analysis used in the study considers prescriptions of different drugs in a binary fashion and does not take absolute dose or changes in dose into account. If a patient was prescribed a medication throughout the period studied but the dose was altered, our statistical model is not fit to notice this. Therefore it is possible that prescription patterns may affect opioid-related mortality in ways that cannot be captured by this study. Additionally, opioids were only analyzed as a group and not individually, so it is possible that the prescription of certain opioids is temporally associated with death. Our study population is in the smaller range since each drug is prescribed to relatively few patients. A similar analysis on a larger population would produce narrower confidence intervals and allow analysis on subgroups, such as cause of death.

Also, the study here was based on patients included because of autopsy detection of a number of classical opioids occurring in many of the drug-related fatalities seen in the present setting. In recent years, a large number of novel opioid and non-opioid (in particular the latter) compounds have arisen in the illicit drug market [57,58]. The inclusion of opioids commonly involved in overdose deaths is likely to exclude such newer substances, although many of these are not opioids and should not be considered here. The detection of new substances is of key importance in the diagnostic work in identifying causes of death, and novel synthetic compounds were not systematically included in the present study. However, the present study specifically aimed to assess opioid-related overdose fatalities, as these represent the vast majority of illicit drug poisonings. Thus, the incapacity of the present study to include all substances potentially involved in drug-related fatalities thereby present a limitation to some extent. Due to the original aims of the present overall project, other opioids than morphine, heroin, buprenorphine, methadone, fentanyl and oxycodone were not included in the study. These substances, however, represent 96 and 92 percent, respectively, of patients dying from opioid poisonings and who have been examined in forensic autopsy [59]. The only opioid reported in previous publications as a cause of death occurring in a substantial number of cases [59] is tramadol, which has emerged as a common drug of misuse among adolescents and young adults in Sweden in recent decades [60]. Despite this, we believe the inclusion of all other opioids involved in fatal poisonings still represent a broad picture of acute opioid overdose-related deaths in the present setting.

In addition, it also should be borne in mind that the present study was conducted prior to the COVID-19 pandemic, and it cannot be excluded that prescription patterns and mortality from licit and illicit drugs may have been altered due to the extensive changes in society during the pandemic. Authors have called for attention to the use of addictive substances in that situation [61]. On the other hand, regional health care data from the same region as we studied here, have demonstrated very modest effects on prescriptions for mental health disorders during the pandemic, even during the most intensively affected periods [62]. While it is beyond the scope of the present study, future research should address how the relationship between prescriptions and drug-related mortality may have changed in regions with lower or higher impact from COVID-19.

The classification of drugs in blood at the time of death as prescribed or not is based on the presence of a filled prescription for the drug within the last six months of life, making it a conservative approach. Therefore, estimates of the proportion of prescription drugs with potential for misuse found in blood at the time of death that were used illicitly should be interpreted as possibly low in relation to actual numbers.

## 5. Conclusion

The widespread prescription of BZD, pregabalin and z-drugs in our study on individuals with a history of illicit substance use who died with at least one opioid present in their blood is cause for concern. These drugs were involved in most opioid overdose deaths in our material and have previously been linked to increased opioid overdose mortality when used in combination with opioids [6–11,13,20]. Further research should therefore explore the causality in prescription of such drugs and increased mortality in this population and determine to what extent the comorbidity that the prescription of these drugs indicates affects mortality when treated with other established interventions. In the meantime, we suggest that physicians be highly cautious about prescribing these drugs to patients who are simultaneously prescribed opioids or have a non-medical use of opioids. We particularly advise against alprazolam in this context, based on previous research discussed earlier and based on our findings that alprazolam is the most common non-opioid substance found in blood in overdose deaths, that it has a high proportion of illicit use, and that both prescription and dispensing of alprazolam significantly and substantially increased the odds of death during the same month.
